# AI literacy in undergraduate medical education: a competency-based interpretive framework for curriculum and assessment

**DOI:** 10.3389/fmed.2026.1871524

**Published:** 2026-07-14

**Authors:** Chao Fu, Jingjing Li, Haoyi Fan, Ruihang Ren, Kefei Cui, Yan Zhang, Jing Yu, Jing Li

**Affiliations:** 1 Department of Ultrasound, The First Affiliated Hospital of Zhengzhou University, Zhengzhou, Henan, China; 2 School of Computer Science and Artificial Intelligence, Zhengzhou University, Zhengzhou, Henan, China; 3 Department of Radiology and Interventional Radiology, The First Affiliated Hospital of Zhengzhou University, Zhengzhou, Henan, China

**Keywords:** AI literacy, competency-based medical education, curriculum development, medical education, programmatic assessment, undergraduate medical education

## Abstract

Artificial intelligence (AI) is becoming a core educational concern in undergraduate medical education as AI-enabled tools increasingly shape clinical workflows, learning environments, and patient care. The challenge is no longer simply whether AI should be included in the curriculum, but how AI literacy should be bounded for undergraduate learners and translated into teachable, observable, and assessable educational outcomes. This focused conceptual narrative review synthesized literature on AI literacy and related constructs in undergraduate medical education, using a structured search and interpretive synthesis with competency-based medical education (CBME) as an interpretive lens. PubMed and ERIC were searched for English-language literature from 1 January 2020 to 15 April 2026. Local screening records identified 94 standardized bibliography records, 66 records screened after deduplication, 40 full-text reports assessed, and 30 publications contributing to the final synthesis. Five recurring domains were identified: Foundational AI knowledge; applied clinical interpretation and use; data literacy and critical appraisal; ethics, law, and professional responsibility; and human–AI collaboration and professional formation. Through a CBME lens, these domains can be translated into learning outcomes, contextualized tasks, observable performances, and programmatic assessment evidence. The literature most strongly supports conceptual clarification, domain identification, and curricular translation, whereas evidence for longitudinal development, observable performance, and validated undergraduate assessment remains limited. The proposed framework, examples, milestones, and rubric anchors are synthesis-informed design propositions that require empirical validation before high-stakes use.

## 1 Introduction

Artificial intelligence (AI) is increasingly being treated as a core educational concern in medical education rather than as a peripheral technical topic (1). Across diagnostic support, medical imaging, pathology, risk prediction, clinical documentation, and patient communication, AI-enabled tools are becoming more visible in real clinical environments rather than remaining peripheral technical innovations (2–8). This creates an educational problem for undergraduate medical education: future physicians will need to understand, question, communicate about, and remain accountable for clinical and educational work in which AI-supported information may shape judgment, workflow, and patient care (9–14).

The rapid diffusion of large language models and other AI-enabled systems has intensified this challenge, although AI literacy in medicine extends beyond generative AI alone (15). Students may encounter AI both as future clinicians preparing for AI-enabled practice and as current learners engaging with AI-supported tools, outputs, or educational environments (10, 16). The focus of this review is AI as an object of learner competence, rather than AI primarily as an instructional technology.

The current literature indicates that undergraduate medical students require more than general awareness or exposure to AI (10, 13, 17–19). However, the educational target remains difficult to specify. Terms such as AI literacy, AI competence, AI readiness, data literacy, and digital health competence overlap across the included publications, and they are not always used with stable boundaries (20–23). At the same time, recent frameworks, consensus studies, Delphi studies, and curriculum-oriented publications have begun to identify AI-related competencies, learning priorities, and curricular domains for medical education (18, 23–26). The field has therefore moved beyond general calls to include AI in the curriculum, but it has not yet fully clarified how AI literacy should be bounded for undergraduate learners or translated into teachable, observable, and assessable educational outcomes.

Competency-based medical education (CBME) provides an appropriate interpretive lens for this translation, as the literature repeatedly identifies competency frameworks, learning outcomes, curricular integration, observable performance, and assessment as central educational concerns (13, 23–27). Applied to AI literacy, a CBME lens shifts attention from whether students have merely been exposed to AI-related content toward whether they can interpret AI-supported outputs, recognize limitations and bias, communicate uncertainty, and exercise accountable clinical judgment in context (9, 13, 14, 22, 28). CBME does not by itself resolve the implementation challenge, but it provides a structure for examining how AI-related learning might be organized, developed, observed, and assessed within undergraduate training.

This focused conceptual narrative review synthesizes how AI literacy have been conceptualized and educationally translated in undergraduate medical education. Rather than offering a broad overview of AI in medical education, it examines how AI literacy has been framed, how its undergraduate boundaries can be defined, and how recurring competency domains can be linked to curriculum, observable performance, and assessment through a CBME lens. Its central contribution is to organize AI literacy as a bounded, competency-oriented construct for undergraduate medical education and to clarify a synthesis-informed pathway for translating that construct into educational design and assessment.

## 2 Methods

### 2.1 Review design

This study was conducted as a focused conceptual narrative review using a structured literature search and interpretive synthesis of how artificial intelligence (AI) literacy has been conceptualized and educationally translated within undergraduate medical education, with particular attention to competency-based curriculum and assessment.

The review aimed to identify recurring ways in which AI literacy and related constructs were framed, linked to educational outcomes, and translated into a CBME-oriented interpretive framework for undergraduate medical education. AI literacy served as the primary organizing construct, while related terms such as AI competence, AI readiness, attitudes, and perceived learning needs were included when they informed its educational meaning, boundaries, or translation, but were not treated as equivalent constructs. The search was structured to support conceptual coverage and interpretive synthesis rather than exhaustive evidence capture.

### 2.2 Information sources, search strategy, and eligibility

A targeted search of PubMed and ERIC was conducted to identify English-language literature published or available from 1 January 2020 to 15 April 2026. Search terms combined concepts related to the learner population, AI-related topics, and educational constructs or outcomes. Complete database-specific search strategies, search dates, and record yields are provided in Supplementary Appendix 1. Broader education terms such as medical education and curriculum were retained to avoid missing publications with clear undergraduate implications that were not indexed explicitly as undergraduate-focused.

Publications were eligible if they were peer-reviewed, written in English, addressed undergraduate medical education directly or had clear implications for the undergraduate phase, and contributed to at least one of the following areas: conceptualization of AI literacy or related constructs; competency domains, learning objectives, or educational frameworks; curriculum initiatives or teaching strategies; student readiness, attitudes, perceived readiness, or learning needs; or approaches to observable performance and assessment. Both conceptual and empirical publications, including quantitative, qualitative, and mixed-methods studies, were eligible. Publications focused primarily on technical model development without substantive educational content, on AI-assisted teaching tools without clear relevance to learner AI literacy, or exclusively on postgraduate or specialist training without transferable relevance to undergraduate medical education were excluded.

Direct undergraduate medical education literature was prioritized. Adjacent literature from health professions education, AI literacy scholarship, digital health competency literature, and CBME theory was included only when it provided contextual or interpretive support for issues insufficiently addressed in undergraduate-specific publications.

### 2.3 Study selection and analytic extraction

Records were screened in two stages, first by title and abstract and then by full-text review. Papers considered potentially relevant were retained if they informed the conceptualization, educational structuring, or assessment of AI literacy or related competencies in undergraduate medical education. Study inclusion decisions and domain classifications were independently verified by a second reviewer. Disagreements were resolved through discussion and, when necessary, consultation with a third author. Because conceptual coding was iterative, non-mutually exclusive, and interpretive, formal agreement coefficients were not used as the sole indicator of analytic credibility.

**FIGURE 1:**
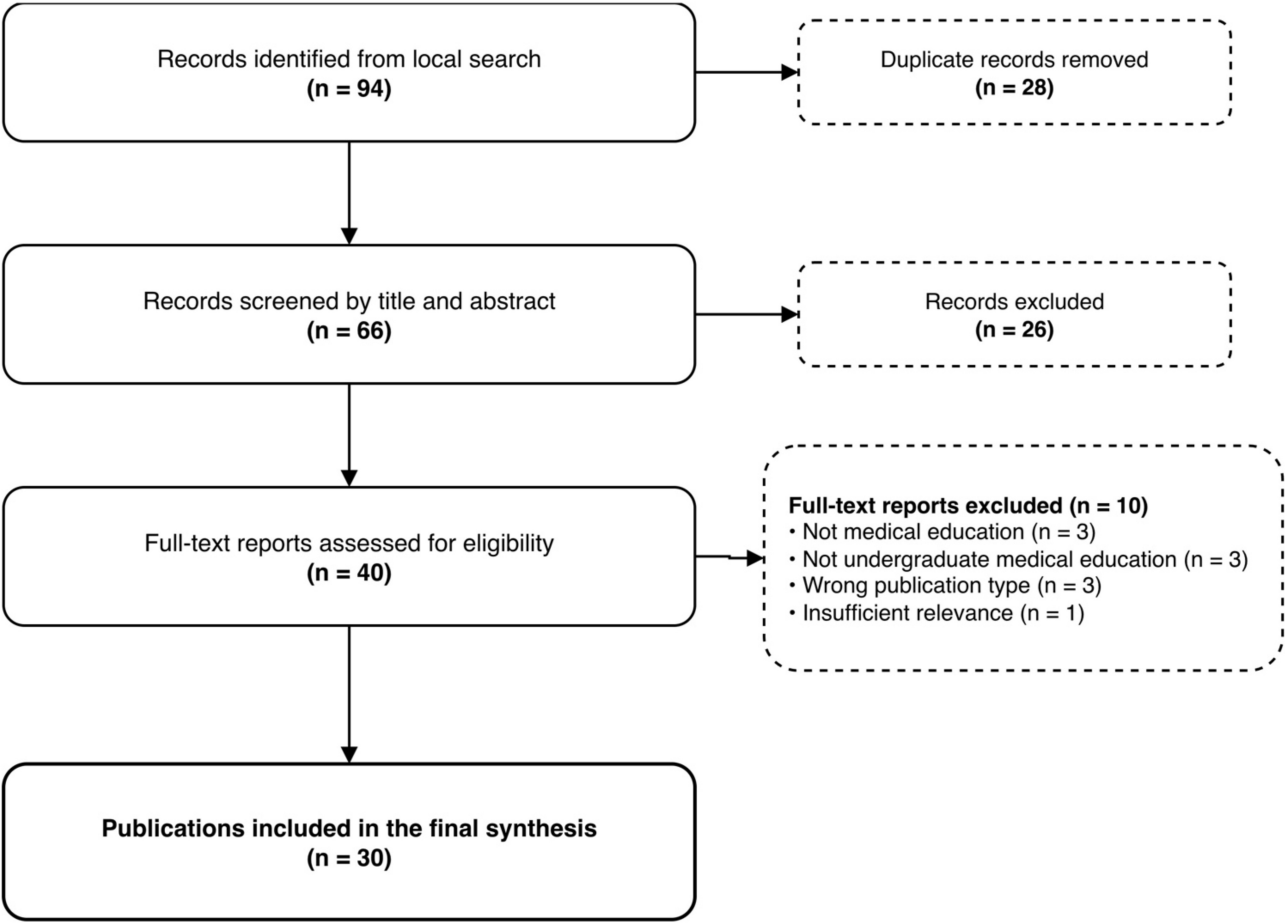
PRISMA-style flow diagram of source identification, screening, and inclusion for the focused conceptual narrative review.

The local search and screening record documented 94 standardized bibliography records. After removal of 28 duplicate records, 66 records were screened by title and abstract; 26 were excluded and 40 full-text reports were assessed. Ten full-text reports were excluded for the following recorded reasons: not medical education (*n* = 3), not undergraduate medical education (*n* = 3), wrong publication type (*n* = 3), and insufficient relevance (*n* = 1). Thirty publications were included in the final synthesis record. The detailed procedure is illustrated in Figure 1.

For each included paper, information was extracted on publication type, educational context, terminology used, stated educational focus, competency-related elements, curricular implications, and any discussion of observable performance or assessment. Extraction focused on conceptual comparison across papers rather than formal evidence grading or intervention-focused tabulation.

### 2.4 Conceptual synthesis

No formal methodological quality appraisal or risk-of-bias assessment was undertaken because the purpose of the review was conceptual synthesis rather than comparative evaluation of intervention effectiveness or estimation of educational impact. Instead, the analysis focused on how constructs, learning outcomes, curricular implications, and assessment ideas were represented across the included and contextual literature.

Through repeated reading and cross-paper comparison, patterns of convergence, overlap, and inconsistency were identified across the literature. During iterative comparison, later publications predominantly elaborated, contextualized, or reinforced existing categories rather than generating additional major domains. We therefore describe the synthesis as achieving conceptual sufficiency rather than formally measured thematic saturation. This judgment describes analytic stability and was not used to stop screening otherwise eligible publications. Supplementary Table 2 provides an audit trail showing how source constructs were consolidated, excluded, or treated as implementation conditions.

### 2.5 Characteristics of the included literature

The included publications were heterogeneous in publication type, design, and evidentiary orientation, with the largest proportion appearing in 2025. They included conceptual and framework-oriented papers, reviews and scoping reviews, consensus or Delphi studies, curriculum reports, and empirical studies examining readiness, exposure, attitudes, learning needs, barriers, or selected educational outcomes.

The analytical contribution of the included literature was unevenly distributed across domains. Because domain coding was non-mutually exclusive, many publications contributed to more than one area. Curriculum design was the most frequent contribution, followed by competency frameworks, implementation barriers, assessment, ethics and professionalism, learning outcomes, conceptualization, and human–AI collaboration. A smaller number of publications contributed specifically to digital health competence or data literacy. This pattern indicates that the literature most strongly supports synthesis of how AI literacy and related constructs have been framed, which competency domains recur, and how AI literacy or AI competence may be translated into undergraduate medical curricula.

By contrast, evidence directly addressing observable performance, longitudinal competence development, or robust assessment in authentic undergraduate medical education settings remained comparatively limited. Although assessment was represented in the included publications, it often appeared as assessment implications, self-reported readiness, preliminary item development, or bounded course evaluation rather than as sustained measurement of demonstrated competence over time. Accordingly, the evidence base allowed stronger synthesis of conceptualization, domain structure, curricular translation, and implementation considerations than of longitudinal assessment or performance-based competence standards. This imbalance informed the review’s interpretive orientation, and the level of certainty attached to different aspects of the synthesis. Supplementary Table 1 summarizes the included publications and their analytical contribution to the present synthesis.

## 3 A conceptual shift in the literature: from content exposure to competency-oriented AI literacy

Across the included literature, AI-related learning in undergraduate medical education was not framed only as exposure to new concepts, applications, or tools. Some publications emphasized familiarity with AI principles and clinical applications as medicine becomes increasingly technology enabled. However, a broader pattern across the reviewed literature framed AI literacy around a competency question: interpretation, critical appraisal, communication, and professional accountability in AI-enabled clinical contexts (11–14, 16, 20, 23, 25, 26, 28). This pattern supports treating undergraduate AI literacy as competency-oriented AI literacy rather than content exposure alone.

**FIGURE 2:**
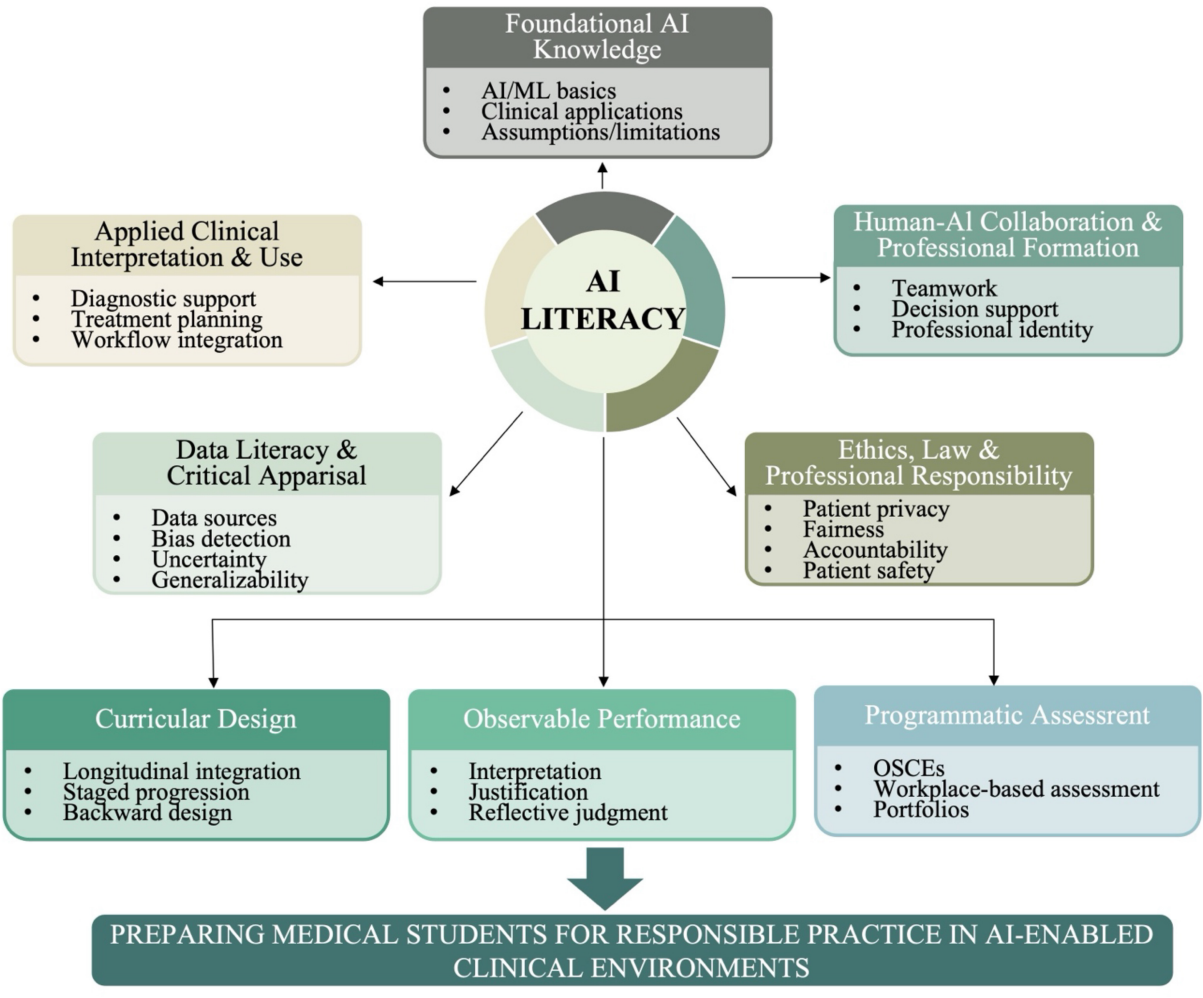
A CBME-oriented framework for AI literacy in undergraduate medical education: core domains and translation into curricular design, observable performance, programmatic assessment, and responsible practice.

CBME provides the organizing lens for translating this conceptual shift into educational design. Its emphasis on explicit competencies, developmental progression, contextualized performance, and aligned assessment helps connect AI literacy with what learners should progressively understand, demonstrate, and be judged on in educational or clinical settings (24, 29). In this review, CBME is used not as evidence that AI literacy has already been operationalized or validated, but as a structure for organizing the synthesis that follows: Section 4 clarifies the construct and recurring domains, Section 5 examines curricular translation, and Section 6 considers observable performance and assessment. Figure 2 summarizes the proposed pathway from AI literacy as a broad construct to competency domains, curricular translation, observable performance, and assessment.

## 4 Conceptualizing AI literacy for undergraduate medical education

### 4.1 Terminological heterogeneity and areas of overlap

No single stable term was used to describe AI literacy and related constructs in undergraduate medical education. Related constructs included AI literacy, AI competence, AI readiness, data literacy, digital health literacy, and informatics capability (11, 30–32). Although these terms overlapped, they were not used consistently or interchangeably. Some emphasized knowledge and understanding, others focused on practical preparedness, and still others foregrounded broader professional or digital capabilities.

This terminological heterogeneity has direct educational consequences because unclear constructs are difficult to translate into curriculum and assessment. When the target of learning remains unstable, it becomes difficult to specify which elements should be considered core, what level of attainment is appropriate for undergraduate learners, and how competence should be recognized in practice.

### 4.2 A working definition for undergraduate medical education

Despite terminological variation, the included literature converged on a common educational concern: AI literacy in undergraduate medical education should support informed, critical, communicative, and professionally accountable engagement with AI in clinically relevant educational and practice contexts (9, 12, 14, 20, 28, 33, 34). For the purposes of this review, AI literacy is operationally defined as the capacity to engage with AI in clinically relevant ways that are informed, critical, communicative, and professionally accountable. This working definition is a synthesis-derived interpretive formulation rather than a standardized definition used uniformly across the literature (21, 27, 34, 35).

AI literacy is used here as the broader educational construct, whereas AI competence refers more specifically to the observable enactment of AI-related understanding, judgment, communication, and professional responsibility in educational or clinical contexts. This distinction allows AI literacy to include conceptual, critical, ethical, and professional dimensions while still requiring translation into competencies that can be demonstrated and assessed.

### 4.3 Recurring competency domains in the literature

The included literature did not present a single standardized taxonomy of undergraduate medical AI literacy. Instead, the included publications contributed partially overlapping emphases across AI literacy conceptualization, competency frameworks, curriculum design, learning outcomes, assessment, implementation barriers, ethics and professionalism, human–AI collaboration, data literacy, and digital health competence (9, 11, 13, 16, 22, 23, 25, 26, 28, 36). The five domains synthesized in this review were therefore derived through convergence across the included literature rather than direct adoption from any single framework.

When AI literacy frameworks, digital health competency publications, consensus and Delphi studies, curriculum-oriented papers, reviews, empirical studies, and ethics-focused commentaries were compared through a CBME lens, several recurrent educational functions became visible. Some publications contributed to multiple areas, and the boundaries between knowledge, skills, attitudes, professional responsibility, and clinical use were not always explicit or mutually exclusive in the original literature. The synthesis below therefore organizes these overlapping emphases into a practical structure for undergraduate medical education, rather than presenting a wholly new competency model.

Five domains were particularly identifiable: Foundational AI knowledge; applied clinical interpretation and use; data literacy and critical appraisal; ethics, law, and professional responsibility; and human–AI collaboration and professional formation (13, 23, 25, 26, 28, 36). Together, these domains capture how AI-related preparedness has been educationally framed for undergraduate medical learners and provide a structure for linking a broad literacy construct with curriculum design, learning outcomes, observable performance, and assessment.

The first domain is Foundational AI knowledge. This domain appeared most consistently in AI literacy frameworks, conceptual analyses, and competency-oriented papers that addressed the definition, scope, and educational purposes of AI literacy (21, 27, 33–35). It refers to a practical understanding of what AI is, how AI systems function at a broad level, and why their assumptions, capabilities, and limitations matter for medical learning and future clinical practice. The emphasis was not on technical mastery, but on helping learners move beyond superficial familiarity with AI tools toward informed recognition of what AI systems can and cannot do.

The second domain is applied clinical interpretation and use. This domain was emphasized mainly in curriculum-oriented, review, and clinically focused publications concerned with how medical students should engage with AI-supported outputs in patient care and educational settings (11, 13, 16, 28, 36). It concerns interpretation of AI-generated information in relation to patient context, other clinical findings, workflow, and decision-making demands. Across these publications, AI use was framed less as technical operation than as contextual clinical reasoning involving appropriate reliance, human oversight, and integration of AI-supported information into broader clinical judgment.

The third domain is data literacy and critical appraisal. Identified across AI literacy frameworks, digital health competency literature, and studies of data science, evidence-informed practice, and AI-supported clinical reasoning (22, 23, 33, 34, 37), it concerns the appraisal of data quality, representativeness, validation, bias, uncertainty, and generalizability. Empirical evidence indicates that students may struggle to evaluate AI outputs: among 148 final-year medical students, the median proportion of correctly evaluated large-language-model responses was 56% (13), while a randomized trial of 111 novice medical students found that plausible but misleading AI explanations reduced diagnostic accuracy (38). Undergraduate outcomes should therefore include detection of both factual and reasoning-level errors, verification of clinically consequential claims, and explicit appraisal of uncertainty and applicability.

The fourth domain is ethics, law, and professional responsibility. This domain was repeatedly foregrounded in ethics-oriented reviews, curriculum papers, professional competency discussions, and consensus-oriented publications. It includes privacy, fairness, transparency, accountability, safety, legal awareness, and the recognition that AI-supported information does not remove professional responsibility.

The fifth domain is human–AI collaboration and professional formation. This domain was most visible in literature addressing future clinical roles, professional identity, interdisciplinary practice, reflective use of AI, and responsible technology adoption (9, 12–14, 39). It reflected concern with changing relationships among clinicians, technologies, teams, and patients in AI-enabled environments. It included reflective judgment, appropriate reliance, adaptability, interdisciplinary awareness, and the capacity to preserve professional responsibility as clinical workflows evolve.

These domains should be understood as interrelated, non-mutually exclusive dimensions of undergraduate AI literacy rather than discrete modules, isolated curricular units, or a validated structure. Their value lies in moving the discussion from a general call for AI literacy toward a structured account of what undergraduate learners should progressively understand, demonstrate, and be assessed on.

### 4.4 Undergraduate boundaries of AI literacy

An important boundary condition across the included literature was that undergraduate AI literacy should be framed around safe, critical, supervised, and professionally responsible participation in AI-enabled learning and clinical environments. The included curriculum papers, reviews, and consensus publications emphasized preparing students to interpret AI-supported outputs, recognizing limitations, engaging with ethical and professional implications, and integrating AI-related judgment into clinical learning, rather than preparing medical students to function as AI engineers or system governors (9, 18, 23, 25, 26, 28, 40). Medical students should therefore not be expected to develop algorithms, undertake advanced model engineering, or assume primary responsibility for system-level AI governance; these activities fall beyond the undergraduate educational boundary implied by the included literature. This boundary keeps the construct educationally realistic while preserving its relevance to future clinical practice.

Defined in this way, AI literacy is broad enough to reflect the realities of AI-enabled medicine while remaining sufficiently bounded for undergraduate CBME. Table 1 summarizes the core domains, their working definitions, clinical relevance, and undergraduate boundaries.

**TABLE 1:** Core domains of AI literacy in undergraduate CBME.

Domain	Working definition	Relevance to practice	Undergraduate boundary
Foundational AI knowledge	Functional understanding of what AI systems are intended to do, where they are used, and where their limits lie	Provides the conceptual basis for safe and critical engagement with clinical AI tools	Oriented to informed use and explanation, not model engineering
Applied clinical interpretation and use	Interpretation of AI-supported output in relation to patient context, evidence, and clinical reasoning	Positions AI within supervised clinical judgment rather than autonomous decision-making	Limited to supervised interpretation, justification, and escalation
Data literacy and critical appraisal	Appraisal of data quality, bias, validation, uncertainty, generalizability, and transferability	Supports judgment about trustworthiness and applicability	Critical reading and verification rather than technical validation
Ethics, law, and professional responsibility	Recognition of privacy, fairness, transparency, accountability, safety, and legal/professional duties	Clarifies that AI does not displace professional responsibility for patient care	Excludes formal governance and regulatory leadership
Human–AI collaboration and professional formation	Reflective, communicative, and professionally accountable engagement with AI in teams and patient-facing contexts	Supports calibrated reliance, teamwork, communication, and professional identity formation	Participation in supervised practice rather than deployment leadership

The domains are analytically distinguished for clarity, although they are interrelated and non-mutually exclusive in practice. They are intended to organize AI literacy within undergraduate competency-based medical education and should not be interpreted as a validated factor structure.

## 5 Translating AI literacy into educational design

The curriculum-oriented literature shifted the problem from whether AI should be included in undergraduate medical education to how AI literacy can be made educationally usable within existing programs (25, 26). Across frameworks, consensus publications, reviews, curriculum reports, and empirical studies, a recurrent translation pathway was identifiable: broad AI literacy domains were converted into learning outcomes, embedded within existing curricular and competency structures, developed through contextualized learning activities, and linked to observable performance and assessment (18, 24, 27, 36).

The first curricular translation task was outcome specification. Broad domains such as data literacy, critical appraisal, and professional responsibility become educationally usable only when expressed as explicit learner expectations. For example, data literacy and critical appraisal can be translated into outcomes such as recognizing dataset bias, questioning model generalizability, or explaining why an AI-supported output may not apply to a particular patient (22, 23). Ethics and professional responsibility can be translated into expectations related to privacy, fairness, accountability, patient safety, responsible use, and communication of uncertainty (9, 11, 12, 34). This conversion from domains to outcomes helps make AI literacy teachable, mappable, and assessable within undergraduate training.

A second translation task was curricular integration. The included literature more often supported embedding AI-related outcomes within existing undergraduate medical education structures than creating a separate parallel curriculum (18, 23, 25, 26, 28). In this integrative model, AI literacy was linked to established areas such as clinical reasoning, evidence appraisal, communication, collaboration, professionalism, ethics, informatics, and practice-based improvement. The emphasis was therefore on aligning AI-related outcomes with existing competency-based curricula rather than treating AI literacy as an isolated technical addition.

This integration can occur both horizontally and developmentally. Horizontally, AI-related learning can be connected with teaching on ethics, evidence-informed practice, informatics, clinical reasoning, simulation, and clerkship or bedside discussion (11, 12, 14, 39). Developmentally, AI-related competencies can be revisited with increasing complexity across undergraduate training, allowing early conceptual understanding to progress toward contextual application, critique, communication, and supervised judgment (17). This staged model is consistent with CBME because it treats AI literacy as a developing professional capability rather than a one-time exposure to AI content.

At the level of teaching practice, the reviewed literature favored active, contextualized, and performance-relevant approaches that make AI-related reasoning visible. Commonly described or recommended approaches included authentic learning tasks, clinically grounded discussion, simulation, reflective practice, practical AI training, evaluation of AI-generated outputs, and competency-driven course activities (12–14, 24, 37, 40). These approaches allow students to rehearse not only knowledge about AI, but also the interpretations, judgments, explanations, and communication expected in AI-enabled clinical contexts. Curriculum reports and empirical studies further suggest that even bounded interventions can address multiple AI-related competencies when explicitly mapped to outcomes and anchored in clinically or educationally relevant tasks (13, 19, 37). Within this staged approach, progression across the five domains may be understood as movement from recognition and explanation, through guided application and critique, toward increasingly independent verification, justified decision making, communication of uncertainty, and appropriate escalation within supervised educational or clinical contexts.

Taken together, these curricular patterns position AI literacy as the staged educational translation of a composite professional competency rather than the addition of a new technical topic. Table 2 summarizes this translation into indicative domain, indicative outcome, learning formats, specialty-agnostic task, observable performance and assessment.

**TABLE 2:** Translating AI literacy domains into curriculum, observable performance, and assessment.

Domain	Indicative outcome	Learning formats	Specialty-agnostic task	Observable performance	Assessment
Foundational AI knowledge	Explain major uses, assumptions, and limits of clinical AI tools	Introductory sessions; guided examples; concept seminars	Review of a brief description of a hypothetical clinical AI tool	Explains what a tool does, where it applies, and where it does not	MCQ/SAQ; case-based written response
Applied clinical interpretation and use	Interpret AI output alongside clinical findings	Case discussion; simulation; AI-supported vignettes	Evaluation of an AI-generated recommendation that conflicts with the patient’s medical history or clinical findings	Accepts, questions, or overrides AI output with justification	OSCE; structured oral case analysis; reasoning task
Data literacy and critical appraisal	Appraise data quality, bias, validation, and generalizability	Critical appraisal sessions; dataset critique; guided paper review	Appraisal of an AI-generated output containing potential bias, insufficient external validation, or hallucinated content	Identifies sources of bias, uncertainty, and limited transferability	Written critique; appraisal assignment; short report
Ethics, law, and professional responsibility	Analyze ethical and professional implications of AI-supported care	Ethics cases; policy discussion; reflective tasks	Analysis of an AI-use scenario involving privacy, fairness, or allocation of responsibility	Recognizes privacy, fairness, accountability, and safety concerns; justifies an appropriate response	Scenario-based response; reflection
Human–AI collaboration and professional formation	Maintain independent judgment and communicate responsibly in AI-enabled settings	Team discussion: simulation debrief; workplace reflection	Communication of an AI-supported conclusion to a patient or healthcare team	Communicates uncertainty, avoids overreliance, and escalates concerns appropriately	OSCE with global rating; portfolio; workplace-based assessment

MCQ, multiple-choice question; SAQ, short-answer question; OSCE, objective structured clinical examination. Observable performance may be interpreted using four illustrative developmental anchors. Level 0 reflects unsafe performance, with uncritical acceptance of AI outputs or failure to recognize key errors or risks; Level 1 reflects emerging performance, requiring prompting and showing incomplete verification or justification; Level 2 represents the expected undergraduate threshold, with independent identification of limitations, verification of key claims, and appropriate decision-making; and Level 3 reflects advanced performance, integrating multiple evidence sources and adapting decisions and communication to context.

## 6 Translating AI literacy into observable performance and assessment

From a CBME perspective, the curricular translation described above is incomplete unless learning outcomes are connected to observable performance and assessment evidence. In the included literature, this connection was addressed less extensively than conceptualization, competency mapping, and curriculum design (25, 26). AI literacy was more often defined, advocated, mapped to competencies, or translated into curricular recommendations than directly assessed as contextualized undergraduate performance (18, 23, 25, 26, 28). The central assessment problem is therefore not simply selecting an instrument, but clarifying what counts as credible evidence that learners can enact AI-related understanding, judgment, communication, and professional responsibility in educational or clinical contexts.

The first assessment task is to specify the performance construct. Across the included publications, observable AI-related competence was described mainly as students’ ability to interpret, justify, critically appraise, communicate, and reflect on AI-supported information in context, rather than as technical operation alone. Assessment-relevant publications pointed toward performances such as explaining the purpose and limitations of an AI tool, evaluating AI-supported outputs, recognizing concerns about bias or data quality, and judging when human clinical reasoning should qualify or override AI-supported information (13, 22, 27, 36, 41). These performances correspond to the domains synthesized in Section 4 and the learning outcomes described in Section 5: foundational conceptual understanding may be observed when a learner explains what an AI tool is designed to do and where its limits lie; data literacy and critical appraisal may be observed when the learner questions dataset representativeness or generalizability; and professional responsibility may be observed when the learner communicates uncertainty and maintains accountability for patient-facing decisions. These performance expectations can also be summarized through the recently proposed Cognitive Integrity Threshold (CIT), defined as the minimum task-relevant understanding required for meaningful oversight, autonomy, and accountable participation under AI assistance (42). Applied to undergraduate medical education, CIT can be interpreted as the minimum ability to explain a tool’s purpose and limits, identify material conflicts, verify consequential claims, justify whether an output should be accepted, qualified, rejected, or escalated, and communicate residual uncertainty.

A second assessment task is to determine the context in which these performances can be observed. Because AI-related competence depends on situated interpretation, the included literature points toward assessment contexts that make reasoning, explanation, and judgment visible rather than relying only on decontextualized recall (11, 13, 24, 36, 39). Relevant examples include evaluation of AI-generated outputs, clinically grounded or case-based discussion, simulation-based learning, competency-driven course activities, reflective approaches, and supervised educational tasks linked to defined outcomes (13, 37, 40). These settings allow educators to observe not only whether students know AI-related concepts, but whether they can apply, explain, and qualify them in relation to clinical or educational tasks. In this respect, assessment becomes a continuation of curriculum design: the same clinically anchored activities that support learning can also generate evidence of interpretation, communication, and professional judgment.

A third assessment task is to distinguish indirect indicators from evidence of demonstrated competence. Several included empirical studies and surveys addressed exposure, readiness, attitudes, perceived learning needs, barriers, confidence, or acceptability, which are useful for describing learner perspectives and implementation needs but are weaker proxies for demonstrated AI-related competence (10, 19, 43). Similarly, AI literacy item sets and assessment-oriented frameworks contribute to construct clarification and measurement development, but they should not be treated as sufficient evidence that learners can enact AI-related reasoning in clinical or simulated contexts (27, 41). Written or survey-based assessments may sample foundational understanding or perceived readiness, but they cannot, on their own, capture situated interpretation, accountable judgment, or communication in context (13, 19, 41).

A fourth assessment task is to align methods with the type of evidence sought. The included literature suggests that different evidence sources are needed for different parts of the construct: foundational understanding may be sampled through written or structured tasks; critical appraisal may be assessed through interpretation of AI-supported outputs or data-related scenarios; clinical use may be assessed through case-based or simulated tasks; and professional responsibility may be assessed through explanation, communication, reflection, or justification of decisions (13, 22, 28, 36, 39). The implication is not that one format should be privileged as the assessment tool for undergraduate AI literacy, but that assessment methods should be selected according to the domain, level of training, clinical context, and type of performance evidence required.

These observations point toward a programmatic rather than single-instrument approach to assessment. AI literacy is a composite construct, and credible judgment about undergraduate AI competence is unlikely to arise from a single test, survey, station, or self-report scale. Multiple forms of evidence are better aligned with the construct because they can capture knowledge, reasoning, appraisal, communication, reflection, and professional accountability across different learning contexts (13, 22, 24, 28, 39). Such an approach is also consistent with CBME, in which assessment supports developmental progression and makes performance expectations explicit rather than producing a one-time measure of content acquisition.

At the same time, this assessment direction should be interpreted cautiously. The included literature supports plausible assessment targets more strongly than it validates specific undergraduate assessment tools or programmatic assessment models (19, 37). Direct evidence remains limited regarding how reliably faculty can judge AI-related reasoning, how rubrics should define developmental progression, how performance should be sampled across contexts, and how students’ AI-related competence changes longitudinally.

Taken together, the assessment-related literature supports a shift from measuring exposure, attitudes, confidence, and perceived readiness toward generating evidence of AI-related reasoning, contextual interpretation, verification, communication, calibrated reliance, and professional responsibility. This alignment is summarized in Table 2. Before these assessment approaches are used for summative or high-stakes decisions, validity evidence should be accumulated for their intended interpretation and use. This should include content evidence from domain-based blueprints and multidisciplinary review; response-process evidence from learner and rater studies; internal-structure evidence from reliability, task consistency, and, where appropriate, generalizability analyses; and evidence of expected relations with clinical reasoning, evidence appraisal, and AI knowledge. Fairness, classification consequences, and standard setting should also be evaluated. These considerations represent a future validation pathway rather than validation undertaken in the present conceptual narrative review.

## 7 Discussion

This review found that the included literature on AI literacy and related constructs in undergraduate medical education has moved beyond general advocacy for curricular inclusion, but remains unevenly translated into clearly bounded undergraduate outcomes, observable performance, and assessment evidence. Across the 30 publications, the strongest areas of contribution were curriculum design, competency frameworks, implementation barriers, learning outcomes, ethics and professionalism, and human–AI collaboration, whereas assessment was more often represented as assessment implications, preliminary item development, readiness surveys, learner perceptions, or bounded course evaluation than as validated undergraduate performance assessment (Supplementary Table 1).

The main contribution of this review is conceptual and translational. It distinguishes undergraduate AI literacy from technical AI expertise, broader digital health competence, and self-reported readiness or attitudes, defining it instead as preparation for informed, critical, communicative, and professionally accountable engagement with AI-supported information in clinical and educational contexts. It then translates this construct into five undergraduate domains and an aligned curriculum-and-assessment pathway (Figure 2).

The mapping between the present framework and AI-PACE is non-exclusive and many-to-many, as the two frameworks operate at different levels of organization (Table 3). Human–AI collaboration and professional formation align particularly with the Affective dimension, while also involving Psychomotor capabilities such as communication, verification, teamwork, and escalation. The Embedded dimension is better interpreted as a longitudinal curriculum principle supporting the progressive integration of all five domains rather than as an additional learner competency. The frameworks are therefore complementary: AI-PACE emphasizes learning domains and curriculum architecture, whereas the present framework specifies undergraduate-bounded competency content linked to observable performance and assessment (44).

**TABLE 3:** Conceptual mapping of the five-domain undergraduate AI literacy framework to the AI-PACE dimensions.

Present framework	AI-PACE dimension(s)	Complementarity and distinction
Foundational AI knowledge	Cognitive	Functional understanding for safe clinical engagement
Applied clinical interpretation and use	Cognitive + Psychomotor	Situated interpretation and clinical justification
Data literacy and critical appraisal	Cognitive + Psychomotor	Explicit focus on bias, validation, generalizability, and uncertainty
Ethics, law, and professional responsibility	Affective + Cognitive	Patient safety, accountability, legal, and professional duties
Human–AI collaboration and professional formation	Affective + Psychomotor	Calibrated reliance, communication, teamwork, and professional identity

The mapping is interpretive and non-exclusive because the five domains and the AI-PACE dimensions operate at different levels of abstraction. The AI-PACE Embedded dimension is addressed in the accompanying text as a cross-cutting principle of longitudinal curricular integration rather than as a separate learner competency domain. The mapping should not be interpreted as empirical validation or formal equivalence of the two frameworks.

The curricular implication is that AI literacy should be integrated into existing undergraduate medical education structures rather than positioned as a separate technical strand. The included curriculum papers, reviews, and consensus publications support linking AI-related learning to established areas such as clinical reasoning, evidence appraisal, communication, collaboration, professionalism, ethics, informatics, digital health, and practice-based improvement (11, 13, 23, 25, 26). This integrative approach allows AI literacy to be developed progressively, beginning with foundational conceptual understanding and extending toward supervised interpretation, critique, communication, and professional judgment in clinical, simulated, or case-based contexts.

The assessment implication is equally important. If AI literacy is treated as a competency-oriented construct, assessment should move beyond knowledge recall, exposure, attitudes, confidence, and self-reported readiness toward evidence of what learners can do with AI-supported information. Relevant evidence may include students’ ability to interpret AI outputs in context, recognize limitations and bias, detect hallucinations or reasoning errors, verify critical claims, communicate uncertainty, justify calibrated reliance, and maintain accountability when AI informs learning or care. The included literature points toward multi-method and developmental assessment, but does not yet provide a validated undergraduate assessment framework. This remains one of the clearest gaps in the evidence base (13, 36, 41).

Implementation will require more than conceptual clarity. The included publications repeatedly identified implementation barriers and practical requirements, including faculty capacity, curricular space, institutional support, suitable learning activities, ethical guidance, and alignment with existing competency structures (11, 16, 43, 45). Institutions will also need locally adaptable cases, simulation or practice-based opportunities, assessment expertise, and governance arrangements that support responsible curricular integration (25, 26, 36). To promote equitable implementation, learning and assessment should not depend on high-fidelity simulation or continuous access to commercial AI systems; paper- or screen-based cases, pre-generated and faculty-reviewed AI outputs, structured oral tasks, role-play, and facilitated case discussion can make learners’ reasoning, verification, communication, and escalation decisions observable. Faculty development is likely to be essential for implementing undergraduate AI literacy curricula. Rather than requiring advanced technical expertise, faculty should understand the intended uses and limitations of AI, recognize bias, hallucinations, and uncertainty, facilitate discussion of verification, appropriate reliance, and professional accountability, and provide feedback on learners’ reasoning and communication (12, 14, 28, 43). Faculty serving as assessors should also be oriented to the assessment construct and rubric, practice scoring benchmark performances, discuss scoring discrepancies, and participate in periodic calibration to limit rater drift. These requirements should be adapted to local curricular, clinical, and resource contexts.

Several limitations should be acknowledged. First, this was a focused conceptual narrative review rather than a systematic review, and it does not claim exhaustive coverage of all relevant studies. Second, the included literature remained conceptually heterogeneous, with overlapping terminology across AI literacy, AI competence, AI readiness, digital health competence, and data literacy. Third, evidence directly addressing observable performance, longitudinal competence development, or validated assessment strategies in undergraduate settings remained limited.

Future research should move from construct clarification toward implementation and validation. Priorities include longitudinal curriculum studies, faculty development models, performance-based assessment design, rubric development, evaluation of students’ reasoning in AI-supported clinical scenarios, and studies examining how AI-related competence develops across undergraduate training. Research should also examine how students communicate uncertainty, negotiate accountability, recognize bias and limitations, and maintain professional judgment when AI-supported information is introduced into patient care or clinical learning environments.

## 8 Conclusion

Overall, the included literature supports understanding AI literacy in undergraduate medical education as a bounded, competency-oriented educational construct oriented toward critical interpretation, clinical judgment, communication, calibrated reliance, and professional responsibility. A CBME lens helps translate this construct into domains, learning outcomes, curricular activities, observable performances, and assessment evidence. The proposed synthesis offers a practical structure for educational design while identifying a future validation agenda for assessment, faculty development, and equitable implementation.
