# Supplementary material for: AI literacy in undergraduate medical education: a competency-based interpretive framework for curriculum and assessment
Source: Front Med (Lausanne). 2026 Jul 14;13:1871524. doi: 10.3389/fmed.2026.1871524 (PMC13407777; doi:10.3389/fmed.2026.1871524)
Supplement: Supplementary file 1 [file Table_1.docx]

Supplementary Material

# Supplementary Appendix 1. Database-specific search strategies

The formal database search for the focused conceptual narrative review was limited to PubMed and ERIC. The search time range was fixed as 1 January 2020 to 15 April 2026, with English-language limits.

1.1 PubMed

( "Students, Medical"[Mesh] OR "Education, Medical, Undergraduate"[Mesh] OR "medical student"[tiab] OR "medical students"[tiab] OR "undergraduate medical education"[tiab] OR "medical school"[tiab] OR "medical schools"[tiab] ) AND ( "AI literacy"[tiab] OR "artificial intelligence literacy"[tiab] OR "AI competency"[tiab] OR "AI competencies"[tiab] OR "AI competence"[tiab] OR "artificial intelligence competency"[tiab] OR "artificial intelligence competencies"[tiab] OR "artificial intelligence competence"[tiab] OR "AI readiness"[tiab] OR "artificial intelligence readiness"[tiab] OR "AI curriculum"[tiab] OR "AI curricula"[tiab] OR "artificial intelligence curriculum"[tiab] OR "artificial intelligence curricula"[tiab] OR "AI learning objective"[tiab] OR "AI learning objectives"[tiab] OR "artificial intelligence learning objective"[tiab] OR "artificial intelligence learning objectives"[tiab] OR "AI educational framework"[tiab] OR "AI educational frameworks"[tiab] OR "artificial intelligence educational framework"[tiab] OR "artificial intelligence educational frameworks"[tiab] ) AND ("2020/01/01"[dp] : "2026/04/15"[dp]) AND English[la] NOT ( "Internship and Residency"[Mesh] OR resident*[tiab] OR residency[tiab] OR "graduate medical education"[tiab] OR "postgraduate medical education"[tiab] OR nursing[tiab] OR nurse*[tiab] OR pharmacy[tiab] OR pharmacist*[tiab] OR dental[tiab] OR dentistry[tiab] )

The search identified 87 articles.

1.2 ERIC

("artificial intelligence" OR "machine learning" OR "deep learning" OR "natural language processing" OR ChatGPT OR "large language model" OR "large language models" OR "generative AI") AND ("medical student" OR "medical students" OR "undergraduate medical education" OR "medical school" OR "medical schools") AND ("AI literacy" OR "artificial intelligence literacy" OR "AI competence" OR "AI competency" OR "AI competencies" OR "artificial intelligence competence" OR "artificial intelligence competency" OR "artificial intelligence competencies" OR "AI readiness" OR "artificial intelligence readiness" OR "AI curriculum" OR "AI curricula" OR "artificial intelligence curriculum" OR "artificial intelligence curricula" OR "AI learning objective" OR "AI learning objectives" OR "artificial intelligence learning objective" OR "artificial intelligence learning objectives" OR "AI educational framework" OR "AI educational frameworks" OR "artificial intelligence educational framework" OR "artificial intelligence educational frameworks" OR "responsible AI" OR "critical appraisal" OR "human-AI collaboration" OR "trust calibration" OR "calibrated reliance" OR hallucination OR hallucinations)

The search identified 7 articles.

# Supplementary Table S1. Characteristics and analytical contribution of included publications (n = 30)

| **Study** | **Country/ setting** | **Publication type** | **Learners** | **Primary focus** | **Evidence orientation** | **Contribution** | **Key relevance** |
| --- | --- | --- | --- | --- | --- | --- | --- |
| Teng et al. (2026) | China; multi-province online, USMLE-style medical knowledge-testing setting | Original research; three-arm parallel-group randomized controlled trial | 111 junior undergraduate medical students before major clinical rotations | Effects of correct versus misleading AI explanations on diagnostic accuracy and confidence calibration | Quantitative experimental evidence of causal effects on immediate performance and metacognitive monitoring | Demonstrates the asymmetric risk of AI misinformation and supports critical AI appraisal and auditing training | Misleading AI explanations degraded accuracy, promoted targeted errors and generated confidence that did not reliably correspond to correctness |
| Kim et al. (2025) | Not applicable; conceptual curriculum-development guidance in undergraduate medical education | Narrative review; twelve-tips curriculum guidance | Not applicable; no participant sample, with undergraduate medical students as the intended learner group | Development and sustainable implementation of longitudinal, competency-based AI curricula in undergraduate medical education | Conceptual and implementation-oriented analysis informed by literature, author consensus, and institutional curriculum experience | Provides a twelve-tip framework spanning curriculum framing, longitudinal structure, institutional resources, authentic learning, competency-aligned assessment, and adaptive evaluation | Positions critical appraisal of AI outputs, ethical reasoning, authentic clinical simulation, and monitoring of learner behavior and patient safety as core safeguards against unsafe AI use |
| Pucchio et al. (2022) | Canada; cross-sectional survey across all 17 Canadian medical schools, with interviews from 11 schools | Original research; mixed-methods study | 475 undergraduate medical students in the analyzed survey and 17 interviewed students, spanning years 1-4 and combined-degree pathways | Medical students' AI knowledge, perceptions, educational exposure, desired learning formats, and barriers to formal AI curriculum | Mixed-methods observational evidence combining a national cross-sectional survey with qualitative interviews | Documents a national mismatch between students' expected need for AI competence and the scarcity of formal instruction, while identifying workshops, lectures, interdisciplinary activities, and curriculum crowding as implementation considerations | Among analyzed respondents, 75.8% reported no formal AI teaching; only 39% could describe core AI concepts and 63% did not understand AI research methods, indicating limited capacity to critically interpret AI evidence |
| Hackl et al. (2026) | Not applicable; higher-education literature synthesis with illustrative mapping of two degree programs | Narrative review; integrative literature review with expert curriculum mapping | Not applicable; no learner sample, with higher-education students as the intended population | Conceptualization and curricular implementation of a comprehensive, progressive AI-literacy framework for higher education | Evidence synthesis of 27 conceptualizations supplemented by preliminary qualitative expert curriculum mapping | Develops the AI Literacy Heptagon, integrating technical knowledge, application proficiency, critical thinking, ethics, social impact, integration skills, and legal-regulatory knowledge across progressive proficiency levels | Defines critical AI literacy as evidence-seeking appraisal of system capabilities, limitations, claims, and context rather than uncritical acceptance of AI outputs |
| Long et al. (2020) | Not applicable; interdisciplinary AI-literacy synthesis for non-technical learners | Scoping review | Not applicable; no participant sample, with non-technical learners across educational contexts as the intended population | Definition of AI literacy and identification of learner competencies and design principles for accessible AI education | Evidence synthesis from an exploratory interdisciplinary scoping review of 150 documents | Provides an operational AI-literacy definition, 17 competencies, and 15 learner-centered design considerations organized around what AI is, can do, how it works, how it should be used, and how it is perceived | Links calibrated trust to understanding AI strengths and weaknesses and calls for learners to question system intelligence, trustworthiness, data, and outputs as critical consumers |
| Ng et al. (2021) | Not applicable; international educational literature on AI literacy | Scoping review | Not applicable; no participant sample, with learners from school, higher-education, citizen, teacher, and professional contexts represented in reviewed studies | Definition, teaching, assessment, and ethical dimensions of AI literacy across educational contexts | Evidence synthesis based on qualitative coding of 30 peer-reviewed AI-literacy publications | Consolidates AI literacy into four linked domains: knowing and understanding, using and applying, evaluating and creating, and ethical responsibility, while mapping teaching and assessment approaches | Argues that safe AI use requires understanding underlying concepts and ethical consequences, not merely operational use, and identifies critical evaluation and creation as higher-order literacy competencies |
| Yi et al. (2021) | Not applicable; conceptual discussion of AI literacy | Conceptual paper | Not applicable; no participant sample, with learners and citizens in the AI era as the intended population | Conceptual definition of AI literacy through its proposed competence of metacognition and purpose of anticipation | Conceptual and theoretical analysis drawing on literacy theory, futures literacy, and education frameworks | Frames AI literacy as learner agency grounded in metacognition, critical social understanding, and anticipation rather than technical tool use alone | Identifies confidence calibration as a metacognitive ability to distinguish what one knows from what one needs to learn and to assess the quality of AI-mediated information |
| Faruqe et al. (2021) | Not applicable; conceptual AI-literacy competency-model proposal | Conceptual paper | Not applicable; no participant sample, with AI consumers, coworkers, collaborators, and creators as intended competency groups | Development of a research pathway from broad AI-literacy frameworks to behaviorally anchored competency levels and validated assessments | Conceptual and implementation-oriented analysis informed by prior literacy models and applied AI experience | Proposes differentiating AI-literacy requirements by user role and operationalizing them through behavioral anchors, multi-level competency models, assessment development, and a coordinated research matrix | Makes calibrated trust observable by specifying behaviors such as checking data provenance before trusting AI, identifying when human interpretation is required, and examining training data for bias |
| Grunhut et al. (2021) | Not applicable; integrative review of undergraduate medical-education literature | Narrative review; integrative review | Not applicable; no participant sample, with undergraduate medical students as the intended learner group | Medical-student attitudes, proposed or implemented AI curricula, and gaps in evidence for undergraduate medical AI education | Evidence synthesis from an integrative review of 39 publications using PRISMA-informed selection and theoretical appraisal | Shows that calls for curricular change greatly outnumber concrete implementation: only two reports described implemented pilot programs and none evaluated a comprehensive institutional initiative | Argues that physicians need explicit training to understand AI capabilities and limitations, interpret outputs, communicate recommendations, and navigate high-stakes ethical scenarios safely |
| Gordon et al. (2024) | International medical-education literature; 68% of 278 publications originated in North America or Europe | Scoping review | Not applicable; reviewed publications covered medical students, residents, fellows, and physicians across undergraduate, graduate, and continuing education | Mapping the forms, use cases, educational contexts, outcomes, perspectives, and research gaps of AI across medical education | Evidence synthesis from a rapid scoping review of 278 original and perspective publications | Maps AI across admissions, teaching, assessment, and clinical reasoning and proposes the FACETS reporting framework to connect AI form, use case, context, educational form, technology, and integration level | Identifies automation bias, over- and under-skilling, inequity, loss of clinical skills, and weak longitudinal evidence as priority risks requiring explicit study and safeguards |
| Charow et al. (2021) | International health-professions education literature | Scoping review | Not applicable; reviewed literature addressed physicians, nurses, radiology technologists, researchers, administrators, and data scientists across undergraduate, postgraduate, and continuing education | Current and proposed AI education programs for health care professionals, including curriculum content, delivery, implementation factors, and evaluation outcomes | Evidence synthesis from a scoping review of 41 publications, including 13 existing programs | Maps existing and recommended AI curricula into cognitive, psychomotor, and affective domains and derives four guiding principles for regulatory support, multidisciplinary design, competency-based progression, and patient-clinician interaction | Shows that implemented and evaluated programs remain sparse, while variable faculty AI literacy, limited infrastructure, and nonstandard outcome measures constrain safe, comparable curriculum implementation |
| Schubert et al. (2025) | Not applicable; conceptual framework for clinician education across health care settings | Viewpoint | Not applicable; intended for medical students, postgraduate trainees, practicing clinicians, and educators | Defining role-dependent AI expertise and stage-specific questions for incorporating AI education across medical training and continuing education | Conceptual and implementation-oriented expert analysis | Proposes basic, proficient, and expert tiers that combine practical application, critical appraisal, ethics, and technical depth, then links them to curriculum redesign, specialty needs, faculty capacity, and continuing education | Frames safe AI use as requiring clinicians to assess tool utility and outputs, understand ethical implications, retain communication and empathy, and adapt training to local clinical roles |
| Rincón et al. (2025) | International undergraduate medical-education literature, with marked regional differences and limited evidence from low-resource settings | Scoping review | Not applicable; reviewed studies concerned undergraduate medical students and medical educators | Integration of AI into undergraduate medical education, emphasizing curriculum development, competency enhancement, and institutional barriers | Evidence synthesis from a thematically analyzed scoping review of 34 studies | Maps global curriculum heterogeneity and proposes a standardized but regionally adaptable model built around transversal digital skills, interdisciplinary learning, ethics, experiential practice, and faculty development | Highlights weak evaluation evidence, small self-reported samples, scarce longitudinal research, and underrepresentation of low-resource settings as barriers to judging whether AI curricula improve clinical reasoning and safe practice |
| Laupichler et al. (2022) | International higher- and adult-education literature, concentrated in North America and East Asia | Scoping review | Not applicable; reviewed publications addressed higher-education students, adult learners, nonexperts, faculty, and professional groups | Definitions, thematic foci, target groups, disciplines, courses, pedagogies, and evaluation of AI literacy in higher and adult education | Evidence synthesis from a scoping review of 30 publications | Provides a target-group-specific map showing heterogeneous definitions and competencies, varied course designs, and a recurring combination of knowledge transfer with hands-on learning | Shows that course effects are usually measured with self-created, nonvalidated instruments, leaving AI-literacy outcomes poorly comparable and supporting the need for a psychometrically validated scale |
| Laupichler et al. (2023) | Germany; online three-round expert Delphi study | Original research; Delphi consensus study | 53 AI-literacy and AI-education subject-matter experts completed the final Delphi round; most were academics or higher-education professionals | Development and preliminary face and content validation of items for assessing nonexperts’ AI literacy | Psychometric development evidence based on iterative expert consensus and content-validity judgments | Produces an openly reported 38-item pool spanning AI knowledge, application, evaluation, risks, bias, data, and ethics as a foundation for a general AI-literacy assessment | Provides content-validity evidence but not a definitive scale: factor structure, reliability, external validity, and real-world applicability still require testing in a larger normative sample |
| Zhong et al. (2023) | Not applicable; internationally framed medical-school curriculum commentary | Commentary | Not applicable; undergraduate medical students are the intended learner group | Practical content priorities for AI-oriented medical education in response to calls for curriculum integration | Expert commentary without primary empirical data | Proposes a curriculum combining hands-on computational understanding, interdisciplinary teaching, probabilistic reasoning, model limitations, data quality and selection bias, and AI law and ethics | Cautions that technical instruction without uncertainty, explainability, dataset representativeness, and ethical governance may foster overconfidence and unsafe interpretation of AI outputs |
| Saroha (2025) | United Kingdom; conceptual discussion of medical-school education | Commentary | Not applicable; undergraduate medical students and future doctors are the intended learner group | Why and how medical schools should introduce responsible, clinically relevant AI education | Expert commentary with implementation-oriented curriculum proposals | Proposes a phased framework from foundational literacy and ethics to hands-on clinical tools and interdisciplinary innovation, supported by assessment, faculty development, adaptable delivery, and minimum competencies | Emphasizes verification of AI outputs, accountability, privacy, algorithmic bias, equity, preservation of clinical reasoning, and the clinician’s right to reject AI recommendations |
| Rees et al. (2025) | Canada; health-professions digital-health education | Viewpoint | Not applicable; intended for health care professionals, including clinicians, administrators, informatics specialists, and public-health practitioners | Using the Quintuple Aim to prioritize and organize digital-health and informatics competencies and assessments | Conceptual and implementation-oriented competency-framework analysis | Maps competencies and assessment strategies to patient experience, provider experience, cost, population health, and health equity, while combining standardized foundations with role- and region-specific modules | Broadens responsible technology education beyond technical proficiency by tying privacy, ethics, interoperability, usability, cultural safety, digital divides, and patient-centered communication to health-system outcomes |
| Car et al. (2025) | International; experts from 79 countries and territories across all six WHO regions and all World Bank income groups | Consensus statement | 211 experts participated in Delphi round 1 and 149 in round 2; most round-2 participants had teaching or research roles and clinical-medicine backgrounds | Development of an adaptable global digital-health competency framework for preregistration medical education | Consensus-development evidence combining a scoping review, expert consultation, two-round modified Delphi survey, consensus meeting, and qualitative feedback | Establishes the DECODE framework with four domains, 19 competencies, 33 mandatory and 145 discretionary learning outcomes, allowing a common global foundation with contextual adaptation | Makes professionalism, ethics, law, equity, patient digital literacy, health information systems, and data science explicit graduate competencies while acknowledging curriculum-space and implementation constraints |
| Çalışkan et al. (2022) | Türkiye; national online three-round e-Delphi study | Original research; Delphi consensus study | 60 of 69 panelists completed round 3; the panel included health professionals, AI and data specialists, law and ethics specialists, academics, and medical students | Consensus on competencies medical graduates need for AI technologies and applications in medicine | Consensus-development evidence from a three-round e-Delphi survey | Produces 23 strong-consensus competencies covering ethical and legal use, health data, AI application and limitations, professional judgment, teamwork, statistics, and data science | Provides a curriculum-development starting point but reflects Turkish participants and expert consensus rather than demonstrated learner performance; cross-country validation remains necessary |
| Hamilton (2024) | United States; conceptual review of health care simulation and medical education | Narrative review | Not applicable; discusses health-professions students, trainees, residents, and simulation faculty | How AI may transform simulated patients, team and procedural training, instruction, curriculum alignment, assessment, and AI literacy | Narrative evidence synthesis and expert interpretation | Integrates AI-enabled simulation, virtual patients, adaptive feedback, procedural assessment, diagnostic decision support, and curriculum redesign into a field-wide account of simulation as an AI training and testing environment | Cautions that gains in targeted competencies can coexist with degraded overall performance and that hallucinations, bias, privacy, opacity, over-reliance, and accountability require simulation-based testing and guardrails |
| Naseer et al. (2025) | Karachi, Pakistan; single tertiary-care medical college | Original research; mixed-methods study | Survey: 236 participants, comprising 153 second- to fourth-year medical students and 83 faculty; qualitative component: three eight-student focus groups and six faculty interviews | Knowledge, attitudes, practices, perceptions, barriers, and implementation strategies for integrating AI into undergraduate medical education | Mixed-methods evidence combining a cross-sectional survey with focus groups and faculty interviews | Documents positive attitudes alongside limited foundational knowledge, informal AI use, faculty-readiness needs, technological and financial constraints, and preference for phased, context-specific curriculum integration | Shows that responsible integration in a resource-limited setting requires faculty development, ethical and privacy safeguards, inclusive access, and explicit protection of critical thinking and clinical judgment from over-reliance |
| Waldock et al. (2025) | United Kingdom; final-year medical students at Imperial College School of Medicine | Original research; cross-sectional survey | 148 final-year medical students | Ability to evaluate GPT-3.5 clinical answers, learning experiences used in evaluation, and awareness of prompt engineering | Mixed-methods evidence combining answer-accuracy measures self-report and qualitative content analysis | Identifies pathology teaching and case-based question practice as the learning experiences students most often considered helpful, while revealing minimal prompt-engineering awareness | Only 56% demonstrated the prerequisite knowledge needed to safely evaluate AI-generated clinical answers after vague or inaccurate justifications were excluded, supporting explicit teaching on factuality bias and rejection of unsafe outputs |
| Solak et al. (2025) | Canada; undergraduate and clerkship medical education | Commentary | Not applicable; medical students, clerks, and future physicians | Grassroots, reflective, and bedside-integrated approaches to medical AI literacy | Expert and student commentary without primary empirical data | Proposes a three-part strategy comprising a national resource repository, formal multimodal curriculum integration, and structured collaboration through student and faculty networks | Frames AI literacy as necessary to prevent over-reliance, recognize bias and black-box limitations, and support ethical equitable appraisal of AI during clinical training |
| Krive et al. (2023) | United States; University of Illinois College of Medicine | Original research; mixed-methods study | 20 fourth-year medical students across two elective cohorts | Feasibility and educational outcomes of a competency-driven four-week AI and analytics elective grounded in real-world clinical scenarios | Mixed-methods educational evidence from quizzes skills assessments and student reflections | Provides preliminary implementation evidence from a constructivist backward-designed elective that teaches AI concepts without requiring programming and supports longitudinal integration of AI competencies | High post-course scores and positive reflections provide preliminary feasibility evidence, but the small self-selected sample, group assessment, and open-book unlimited-attempt quizzes limit claims about independent mastery |
| Zheng et al. (2025) | Not applicable; China-based review of psychiatry and medical education | Narrative review | Not applicable; medical trainees and future psychiatrists | Effects of AI on psychiatric theory, diagnosis, treatment, education, ethics, and governance | Narrative evidence synthesis based on a structured multidisciplinary literature search | Provides a multilevel roadmap linking AI-enabled changes in psychiatric classification mechanisms diagnosis and treatment to curriculum reform and governance | Recommends integrating technical and data competencies with ethics empathy humanistic care accountability privacy and bias awareness so future psychiatrists can use AI without weakening professional judgment |
| Pupic et al. (2023) | Not applicable; systematic review of international undergraduate medical education literature | Systematic review | Not applicable; 34 included studies covering medical students, medical professionals, and AI-education recommendations | Current evidence-based recommendations for incorporating AI education into undergraduate medical curricula | Evidence synthesis using PRISMA-guided searching quality appraisal and thematic analysis | Synthesizes six recurring curriculum themes: ethics, theory and application, communication, collaboration, quality improvement, and perceptions and attitudes | Supports a standardized clinically grounded curriculum that teaches critical appraisal interpretation and communication, while showing that heterogeneous studies and absent longitudinal evaluations leave effectiveness uncertain |
| Singla et al. (2024) | Canada; national expert panel developing an undergraduate medical curriculum | Original research; Delphi consensus study | 18 Canadian subject-matter experts in health, medicine, education, and AI | Essential AI learning competencies and feasible integration strategies for Canadian undergraduate medical education | Quantitative consensus evidence from a three-round Delphi process with expert ratings and sensitivity analyses | Identifies 82 of 107 competencies as essential, with unanimous early consensus on ethics communication collaboration and quality improvement, and maps them to Canadian EPAs and CanMEDS roles | Prioritizes validation, strengths, limitations, safe use, and clinical interpretation over programming-intensive content, enabling integration into existing biostatistics case-based learning and clinical rotations |
| Lee et al. (2024) | South Korea; nationwide surveys involving medical schools across the country | Original research; mixed-methods study | 1,174 medical students, 781 faculty members, 28 medical AI experts, and 33 medical educators | Definition and prioritization of AI competencies required of South Korean medical graduates | Mixed-methods evidence combining Delphi consensus content analysis nationwide questionnaire data and psychometric factor analysis | Develops a six-domain framework containing 36 competencies and distinguishes foundational competencies for all graduates from advanced data-science and research competencies that may remain optional | Prioritizes digital-health concepts, basic AI principles, ethics, law, clinical appraisal, and medical-data literacy while preserving professional values, empathy, critical thinking, communication, and collaboration |
| Wood et al. (2021) | United States; Medical College of Georgia at Augusta University | Original research; cross-sectional survey | 117 medical students and 44 clinical teaching faculty with analyzable responses | Medical student and faculty AI awareness attitudes and curriculum interests before integrated AI teaching | Quantitative observational evidence based on a single-institution needs-assessment survey | Documents limited baseline AI understanding but broad interest, including different priorities for students and faculty, and supports longitudinal multidisciplinary curriculum integration | The findings justify faculty development and flexible curriculum design, but single-institution voluntary participation and self-selection limit generalizability |

1. Supplementary Table S2. Analytic audit trail for consolidation into five domains

| **Source construct/term** | **Representative source(s)** | **Initial analytic category** | **Final domain** | **Consolidation or exclusion rationale** |
| --- | --- | --- | --- | --- |
| AI basics; capabilities; limitations | AI literacy frameworks and curriculum papers | Foundational knowledge | Foundational AI knowledge | Merged as functional understanding needed for safe engagement, not technical development |
| Clinical use; decision support; interpretation | UME curriculum and clinical AI education papers | Clinical application | Applied clinical interpretation and use | Consolidated around situated interpretation and justification in supervised contexts |
| Data quality; bias; validation; generalizability; uncertainty | Digital health, AI literacy, and assessment papers | Critical appraisal / data literacy | Data literacy and critical appraisal | Grouped because all concern trustworthiness and transferability of AI-supported information |
| Privacy; fairness; accountability; safety; law | Ethics, Delphi, and competency publications | Ethical/professional responsibility | Ethics, law, and professional responsibility | Grouped as patient-safety and professional-duty concerns |
| Communication; teamwork; appropriate reliance; professional identity | Human–AI collaboration and professional formation literature | Collaboration / professional identity | Human–AI collaboration and professional formation | Consolidated as relational and identity work in AI-enabled practice |
| Model engineering; algorithm development | Technical AI literature | Technical expertise | Excluded / outside undergraduate boundary | Beyond the expected scope of undergraduate medical competence |
| Procurement; institutional governance; deployment leadership | Implementation and governance literature | Implementation conditions | Excluded as content domain | Treated as institutional context rather than a student competency domain |
| Knowledge-skills-attitudes-ethics; AI-PACE; AI literacy heptagon; digital health models | Alternative taxonomies | Alternative structures | Used for comparison | Considered complementary but not adopted as the primary content-domain structure |
